# Getting the genome in shape: the formation of loops, domains and compartments

**DOI:** 10.1186/s13059-015-0730-1

**Published:** 2015-08-10

**Authors:** Britta AM Bouwman, Wouter de Laat

**Affiliations:** Hubrecht Institute – KNAW and University Medical Center Utrecht, Uppsalalaan 8, 3584 CT Utrecht, The Netherlands

## Abstract

The hierarchical levels of genome architecture exert transcriptional control by tuning the accessibility and proximity of genes and regulatory elements. Here, we review current insights into the trans-acting factors that enable the genome to flexibly adopt different functionally relevant conformations.

## Introduction

### Getting access to hidden functionality in a compacted genome

In its untangled, unfolded and completely linearized state, the human genome has a length of ~2 m. To fit it into a nucleus with a diameter of ~10 μm, DNA is wrapped around histone octamers, creating strings of nucleosomes that can be further organized into higher-order levels of compaction [1]. The histone octamer obstructs sequence access for most other proteins, which impairs nuclear processes such as transcription [2]. Binding of sequence-specific transcription factors and associated chromatin-modifying enzymes can induce post-translational modification of histone tails and can facilitate nucleosome removal [3–5], which can turn functional sequences such as promoters and enhancers into active, nucleosome-depleted sites [2]. During development, accessible regulatory sites are created de novo, propagated, or eliminated, and each of these processes is highly regulated [6, 7]. Dynamic competition between chromatin components and trans-acting factors for access to DNA sequences allows considerable fine-tuning of transcriptional output [8, 9], which is essential for developmental decisions and functional complexity [10, 11].

### The importance of hierarchical genome structures for gene regulation

To exert stimulatory or repressive effects on transcription, accessible regulatory DNA elements must be in close spatial proximity to susceptible genes. Enhancers promote transcription by providing a binding platform for transcription factors [12] that can act on (distal) target genes through three-dimensional chromatin looping [13–15]. Most of these loops occur within the boundaries of tissue-invariant topologically associating domains (TADs) [16–19]. TADs are megabase-sized chromosomal regions that demarcate a microenvironment for genes and regulatory elements to roam around in to make productive DNA–DNA contacts [20, 21]. Sequences within a TAD not only find each other with high frequency [16] but they generally also show TAD-wide concerted histone chromatin signatures [16, 17], expression levels [22, 23], DNA replication timing [16, 24], lamina association [16], and chromocenter association [25]. Hence, TADs are believed to represent structural chromosomal units that are of functional importance for the regulatory cross talk that determines gene expression programs.

Chromosomes are structured such that domains with a similar chromatin signature cluster spatially, a phenomenon first appreciated by traditional microscopy studies. Centromeres and flanking pericentromeric repeat regions of different chromosomes aggregate and form microscopically visible chromocenters in interphase nuclei [26]. Similarly, the large ribosomal RNA gene clusters that reside on different chromosomes manage to find each other in almost every cell nucleus to form another easily discernable nuclear entity, the nucleolus [27–29]. The more than one thousand different olfactory receptor genes that lie together in large clusters on nearly every chromosome tend to aggregate in the nucleus in different cell types [30–33], which might reflect a chromatin-specific, rather than gene-specific, clustering. Furthermore, chromosomal regions bound by polycomb group (PcG) proteins and marked by the corresponding trimethylation on lysine 27 of histone 3 (H3K27me3) modification spatially aggregate to form nuclear entities also referred to as polycomb bodies [34–36].

Studies using a derivative of chromosome conformation capture (3C) known as ‘Hi-C’ have revealed that long-range genomic contacts segregate TADs into an active (A) and inactive (B) compartment [37]. Based on recent evidence, these have been divided further into two A and four B subcompartments with distinct chromatin signatures, including a polycomb-enriched subcompartment [18]. The nuclear lamina, which coats the inner nuclear cell membrane, represents a major repressive environment in the nucleus. Correspondingly, the lamina mostly recruits TADs of the B compartment, whereas TADs of the A compartment occupy more central nuclear positions in general. Although lamina association is not incompatible with transcription by nature [38], lamina-associated domains (LADs) tend to be largely devoid of transcription [39], and forced association to the lamina can induce gene silencing [40–42]. In addition to peripheral positioning, TADs in the B compartment also prefer to associate with chromocenters. Recently, this was suggested to be not the consequence of active recruitment of TADs, but of preferential diffusion of chromocenters to the peripheral sites that are also favored by B-compartment TADs [25]. Although artificial recruitment to chromocenters can repress transcription [25, 43, 44], there are several examples of chromocenter-associated genes that are actively transcribed [25, 45]. In summary, while the functional significance of enhancer-promoter loops is undisputed and it is clear that (intra-)TAD structures can provide a three-dimensional frame to direct and facilitate these interactions, the importance of inter-TAD contacts and other higher-order topological features for genome function appears more ambiguous and is less well understood.

**Fig. 1:**
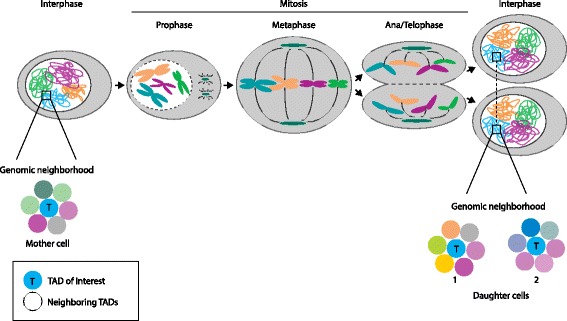
Cell-to-cell variability in genomic neighborhoods. The *upper half* shows a simplified overview of chromatin behavior during the cell cycle. Chromosome territory positioning differs between mother cell and daughter cells (but can be fairly similar between two daughter cells owing to symmetric spindle positioning). In the *lower half*, the zoom view schematically shows the high levels of variation between the genomic neighborhoods of a given topologically associating domain (TAD) of interest (indicated in *blue*) across the mother cell and the two daughter cells 1 and 2. TADs are represented by *colored spheres*

To evaluate these issues, it is important to keep in mind how chromosome folding changes during cell division. Spatial genome organization is generally studied in non-synchronous cells, of which interphase cells make up the biggest proportion. In interphase nuclei, chromosomes are decondensed and organized hierarchically into the transcriptionally relevant structures described above. To prepare for cell division, chromosomes untangle and condense, while transcription ceases almost entirely. Mitotic chromosomes no longer show preferential higher-order contacts or compartmentalized TAD-based organization [46], and it is suggested that enhancer-promoter looping is absent as well [47–50]. Shortly after cell division, chromosomes decondense and reposition themselves in a stochastic manner (Fig. 1), implying that genome topology is not passed down to daughter cells in a precise way. Although individual genes are relatively mobile during early G1 phase, they become quickly constrained to a small nuclear subvolume, after which genome folding is relatively stable for the rest of interphase [51–53].

As can be expected from stochastically reshuffled chromatin, inter-TAD and inter-chromosomal contacts showed high levels of variation between cells in a single-cell Hi-C experiment [54]. Moreover, tracing experiments during cell divisions demonstrated that many of the regions that are positioned peripherally in the mother cell adopt more central nuclear positions in daughter cells, and vice versa [55]. Higher-order genome structures are thus highly variable between otherwise identical cells, with individual TADs adopting different genomic neighborhoods, different positioning relative to nuclear landmarks, and different radial positioning between cells (Fig. 1) [29, 56, 57]. As a result, specific inter-TAD contacts within and between chromosomes [36, 58] are relatively scarce in a cell population. Although they might give rise to cell-to-cell variability in gene expression and could drive changes in cellular identity [59], they cannot be important for its maintenance [56]. Genomic neighborhoods, by contrast, can contribute to this, as discussed below [60]. With all of the above in mind, we will now explore the factors that shape the three-dimensional genome.

## Structuring TADs—the functional units of chromosomes

A chromatinized DNA fiber is assumed to behave essentially as a polymer, with a certain flexibility that allows random collisions between regions of the chromatin fiber. The likelihood for two sites to autonomously find each other in nuclear space decreases when their linear distance increases [37, 61]. The conversion of random chromatin collisions into more stable and potentially relevant structures is assumed to be mediated by interactions between chromatin-associated proteins.

**Fig. 2:**
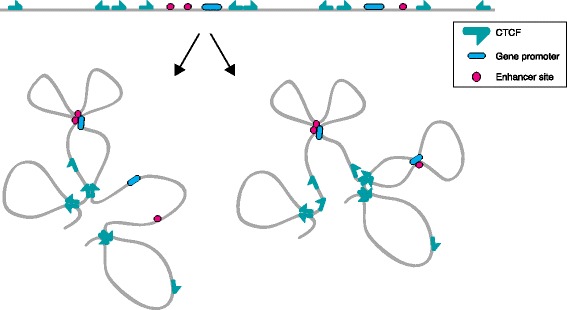
Convergent CTCF sites at topologically associated domain (TAD) boundaries. The linear distribution of CTCF binding sites and regulatory elements across a hypothetical chromosomal segment (*top*) results in three-dimensional looped configurations (*bottom*) that will differ between cells and change over time. CTCF-mediated loops can create TADs, within which enhancer-promoter loops are formed. Loops preferentially occur between convergent CTCF sites, which predicts that a TAD boundary needs to have divergent CTCF sites to accommodate looping with its neighboring boundaries. Note that not all CTCF sites form loops, even when associated with CTCF

The loops formed between TAD boundaries seem to exemplify the longest-range contacts that are stably and reproducibly formed between specific pairs of sequences. Although the mechanisms that underlie the looping of TAD boundaries are largely unknown, numerous reports have identified transcriptional repressor CTCF and the cohesin complex at the sites that anchor these loops [16, 18, 62]. This is in line with previous studies that characterized CTCF at sites separating active and repressed chromatin [39, 63, 64], and that identified both CTCF and cohesin at sites anchoring long-range chromatin contacts [30, 65–68]. CTCF can form dimers in vitro and in vivo [69], and two CTCF molecules bound to distal genomic sites might therefore have the autonomous capacity to form chromatin loops. CTCF has a relatively long non-palindromic DNA recognition sequence [18, 70], and a recent genome-wide assessment of CTCF-bound chromatin loops revealed a strong preference for loops formed between convergently oriented CTCF binding sites (Fig. 2) [18]. The lower efficiency of chromatin looping between CTCF molecules of different orientations could suggest that there is not much intramolecular structural flexibility to accommodate stable long-range interactions, either in the CTCF protein itself or in the chromatin template. Furthermore, if CTCF binding polarity is indeed important for looping, one might expect to find divergent CTCF sites at TAD boundaries because they otherwise cannot capture their two flanking domains in independent loops. In agreement with this, a recent study suggested that diverging CTCF sites represent a general signature of TAD borders in mammals as well as in deuterostomes [71].

**Fig. 3:**
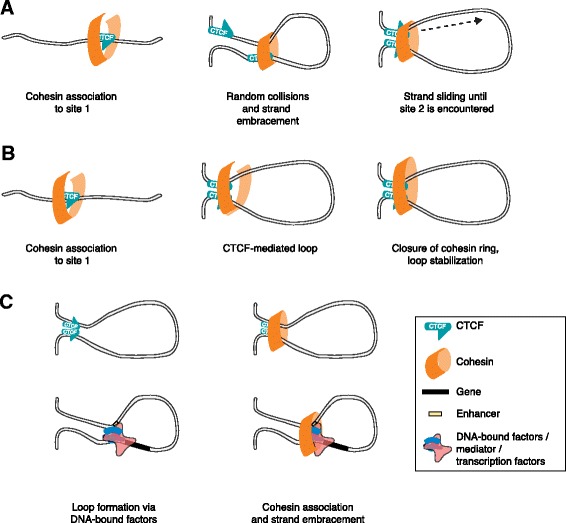
Different scenarios for cohesin-mediated chromatin looping. Three hypotheses for the strategy by which the cohesin complex is involved in the formation of chromatin loops. **a** After initial association of cohesin to one roadblock (such as CTCF), cohesin holds on to this site, and the flanking chromatin is pulled through until a second roadblock is encountered. **b** The cohesin ring remains open when the complex is attached to one roadblock. Only when a second cognate anchor sequence comes in close proximity does the ring close efficiently. **c** Cohesin embraces the DNA anchors of a loop that are already held together by other proteins (*left-hand cartoons*); its embrace stabilizes maintenance of the loops (*right-hand cartoons*)

Cohesin is a protein complex that forms a large ring-like structure to hold the sister chromatids together after DNA replication. In recent years, cohesin has also been found to bind to chromatin in post-mitotic cells [72–74]. Cohesin associates with chromatin at random locations and is thought to slide along the chromatin template. For stable positioning, cohesin relies on chromatin-bound factors, such as CTCF, which might serve as “roadblocks” when bound to chromatin [72]. Cohesin was indeed found to co-associate often at sites occupied by CTCF, but was in addition identified frequently at enhancer-promoter loops bound by the transcriptional coactivator known as mediator [67]. Cohesin might contribute to, or be responsible for, chromatin loops through its ability to embrace two double-stranded DNA helices, supporting an attractive model for cohesin in chromatin organization. How cohesin reaches and grabs the second defined anchor sequence of the to-be-established chromatin loop remains to be determined. One scenario involves a cohesin ring holding on to one associated factor or roadblock, while the flanking chromatin template is pulled through the ring until another roadblock is encountered (Fig. 3a). Alternatively, one can speculate that efficient closure of the cohesin ring only occurs when a cognate anchor sequence with associated factors comes into close physical proximity (Fig. 3b). A third possibility is that cohesin only associates after initial engagement, mediated by CTCF, mediator, and/or transcription factors, to embrace and further stabilize a long-range contact (Fig. 3c). In any of these scenarios, it would be interesting to find out whether cohesin adopts a preferred position upstream or downstream of the oriented CTCF binding site or other cohesin-recruiting roadblocks.

Depletion of CTCF through knockdown resulted in an increased inter-TAD contact frequency, whereas intra-TAD contact numbers were reduced [75]. By contrast, depletion of cohesin subunits led to a more general decompaction and loss of local loops, whereas TADs remained intact [75, 76] or largely intact [68]. Disruption of individual CTCF binding sites was shown to be sufficient to scatter regulatory activity and trigger de-regulation of adjacent genes across TAD boundaries [23]. Insulation of super-enhancer domains or polycomb domains, which generally encompass sub-TAD regions, was released by removing the CTCF sites that demarcate them [77]. Furthermore, removal of CTCF binding sites at a TAD boundary within the *Hox* gene cluster allowed active chromatin marks to expand into a normally repressed domain [78]. In summary, CTCF and cohesin both contribute to definition of TAD boundaries, which appear important for delimiting regulatory influence as their disruption can unsettle local transcription.

## Stabilizing pre-established and de novo formed enhancer-promoter loops

Currently, two types of enhancer-promoter loops are distinguished: pre-established loops and loops formed de novo, or permissive and instructive conformations, respectively [79]. Pre-formed loops provide physical proximity of genes and their cognate regulatory elements irrespective of their transcriptional status, which is believed to facilitate a timely response to developmental stimuli [80–85]. The sonic hedgehog *Shh* gene and its posterior limb-bud enhancer, which are located 1 Mb away from each other at opposite ends of a TAD, exist in such a pre-formed looped configuration that is stable throughout development and that is maintained even after deletion of the enhancer [86]. Mutations in the enhancer that allow recruitment of unrelated transcription factors were shown to expand *Shh* expression to ectopic sites elsewhere in the developing limb bud [85], demonstrating the permissiveness of this preconfigured structure. In general, enhancer sequences are exposed and activated in a highly tissue-restricted manner [19, 87]. Therefore, it remains an open question how preformed enhancer-promoter loops are maintained in unrelated tissues that lack the transcription factors necessary for activating the enhancer elements. We speculate that this is explained by CTCF binding to constitutively looped enhancers [18]. Bookmarking by CTCF, as described below, could contribute to constitutive looping, without necessarily yielding the typical enhancer signatures such as hypersensitivity [87] or histone acetylation [19] in unrelated tissues. Interestingly, even presumed pre-existing configurations might be more dynamic than anticipated. Regulation of transcription mediated through glucocorticoid receptor (GR) signaling involves long-range contacts between GR-bound regulatory elements and target genes. Recently, glucocorticoid treatment was found to alter chromatin accessibility at regulatory sites. Variations in treatment not only correlated with variable life spans of this accessibility, but also with the level of p300 binding and the frequency of regulatory enhancer-promoter loops. This dynamic interplay between transiently altered accessibility and three-dimensional genome organization suggests that we not only need to qualitatively, but also quantitatively, assess looping frequencies and dynamics in order to understand how structure impacts on gene regulation [88].

A recent study provided a first systematic insight into the genome-wide pervasiveness of each loop type across a series of cultured cell lines. While the majority of loops appeared conserved among most of the assessed cell lines and between species, hundreds of tissue-specific enhancer-promoter loops were also uncovered, which nearly always corresponded with strongly increased transcriptional output of the gene involved [18]. Formation of tissue-specific enhancer-promoter loops depends on the association of tissue-specific transcription factors that often recruit ubiquitous factors such as mediator, cohesin, and cohesin cofactor Nipbl that might help establish chromatin loops [67, 89]. Several studies have shown that enhancer-promoter loops dissolve upon depletion of the associated tissue-specific transcription factors [90, 91], which was also usually found to be accompanied by decreased transcription of the target gene. Of note, the inhibition of transcription itself has no impact on the maintenance of chromatin loops [92, 93]. Whether the transcription factors enable loop formation through the recruitment of other protein complexes, such as cohesin, or whether they act as self-associating bridging molecules themselves is currently not clear. Hints that support the latter scenario come from experiments in which the formation of enhancer-promoter loops was forced by employing artificial zinc fingers fused to protein-dimerization domains, which were found to be sufficient to drive loop formation and initiate transcription, even from a stringently silenced gene [94, 95].

## The clustering of TADs with similar signatures

Factors such as CTCF and cohesin seem to be major contributors to the formation and architecture of TADs, whereas other factors appear to be involved in the segregation of TADs into nuclear subcompartments containing similar types of chromatin domains. The rules that govern the relative positioning of TADs in the interphase nucleus must be considered in the context of the genome adopting an energetically favorable conformation upon unfolding after exit from mitosis. Because TADs are parts of much larger chromosomes, the engagement of stable inter-TAD contacts by one TAD imposes constraints on the sampling space of its neighboring TADs. Some regions, in particular the (peri-)centromeric parts of the chromosomes and the ribosomal DNA (rDNA) gene clusters, appear relatively dominant in determining their preferred genomic neighborhood because they find each other in nearly every cell. Others, which might include the olfactory receptor gene clusters and the PcG-bound regions, also retain some autonomy in choosing partners to contact in the nuclear space. Because of this, most of the remaining TADs (and genes) must passively adapt to the resulting configurations [56]. In a process that follows the principles of self-organization [56, 96], the formation of nuclear subcompartments might involve a phase during which TADs scan the signatures of the domains they are spatially surrounded by to position themselves next to chromatin of a similar type. In such a hierarchical positioning process, compartments might also arise not because of particular affinities between TADs that are involved, but merely because they are expelled from other compartments.

Switches between A and B compartments occur for at least a third of the genome during early development, often in a lineage-restricted fashion [97]. The observation that these transitions coincide with only a subtle shift in transcriptional output [97] is in line with previous demonstrations that the act of transcription per se is not required for spatial segregation of active and inactive chromatin [93]. In a recent study, forced activation of endogenous genes with synthetic transcription factors linked to a transcriptional activator prompted repositioning of the loci towards the nuclear interior. Local chromatin decondensation by an acidic peptide was shown to be sufficient to induce comparable spatial repositioning, while the transcriptional state was left unaltered [98]. Thus, chromatin composition and associated trans-acting factors might be the key determinants that control not only transcriptional activity but also the nuclear positioning of TADs. Transcriptional activity and nuclear positioning often correlate but are not expected to directly determine one another. Instead, they could reinforce each other’s states: nuclear subcompartments containing chromatin of similar types will result in local accumulation of the corresponding trans-acting factors, which might facilitate the maintenance of the transcription levels of the associated chromatin.

If not transcription, what is it that keeps active TADs together? Principles similar to those underlying local chromatin loop formation might well drive spatial juxtapositioning of TADs and set up tissue-invariant as well as tissue-specific higher-order topologies. Promoters marked by trimethylation on lysine 4 of histone 3 (H3K4me3) co-localize not only within TADs but also in the larger nuclear space in a largely tissue-invariant manner [16, 23, 99, 100]. Enhancers act in a more tissue-restricted manner and are correspondingly found to be engaged in tissue-specific inter-TAD contacts with other enhancers [100]. Studies of the pluripotent genome uncovered three-dimensional clustering of high-density binding sites for pluripotency factors Oct4, Sox2, and Nanog (which are collectively referred to as OSN), which was hypothesized to boost maintenance of cellular identity [35, 100–102]. In another study, Sox2 enhancer sites were found to form three-dimensional enhancer clusters that optimize the target search dynamics of Sox2 [103]. Furthermore, targeting of Nanog to an ectopic landing platform created novel contacts with OSN binding sites on the same chromosome [100]. Together, these studies illustrate how stage-specific transcription factors can play a direct role in functionally relevant higher-order genome folding. This phenomenon is not specific to the malleable genome of stem cells—a study of the three-dimensional genome during T-cell differentiation revealed that STAT-binding sites aggregate globally in a lineage-specific manner [104]. Transcription factors and other chromatin-associated molecules, including noncoding RNA [93, 105], thus seem to be responsible for inter-TAD contacts and, consequently, formation of subnuclear compartments. Again, these factors likely create such configurations through self-association, mediated by protein complexes bound to two dispersed genomic sites, or through association with histone modifications at both sites. A recent study that was mentioned above also revealed that the artificial recruitment of an isolated HP1-derived chromodomain to a genomic site was sufficient to reposition the region to chromocenters, which was presumed to be owing to an interaction between the chromodomain and modifications involving trimethylation on lysine 9 of histone 3 (H3K9me3) that decorate pericentromeric heterochromatin [25].

## Conclusions and perspectives

Loops, domains, and compartments define the shape of the genome, and all topological levels contribute to the functioning of the genome. Domain organization seems conserved and exhibits an invariance that is remarkable given the observation that TADs are not detected during mitosis [46]. Despite the removal of most chromatin-associated proteins in prophase, it has been suggested that several key regulators, such as CTCF and transcription factors, are retained at a subset of sites during mitosis [50, 106–111]. The rapid emergence of de novo structural organization during early G1 might be driven by mitotically bookmarked TAD boundaries [112] or regulatory elements [113], or by elements marked by DNA methylation or histone modifications [47]. Because most organizational features are believed to derive during early G1 from self-assembly that is guided by local chromatin features, passing on of some information through mitosis potentially results in reproducible local structures, yet increasingly stochastic higher-order assemblies [47]. Indeed, the compartments of different cell types have been reported to vary considerably, which, as discussed, could well contribute to transcriptional fine-tuning and therefore be functionally meaningful. Although evidence suggests that the majority of enhancer-promoter loops are tissue invariant [18], we still need to get a feeling for their dynamics, which might vary more than anticipated between cells and cell types [88]. Future research should therefore aim to visualize the dynamics of enhancer-promoter loops, for example by live-imaging of loop dynamics using advanced high-resolution microscopy methods.

At each level of structural organization, chromatin-associated factors shape the genome. CTCF and cohesin play important roles in chromatin looping—they anchor loops that create chromosomal domains (TADs) and loops that recruit enhancers to their target genes. CTCF might exert its action through self-dimerization or through recruiting cohesin. The observation that loops preferably form between convergent CTCF binding sites (Fig. 2) might have profound implications for our understanding of the flexibility of the chromatin fiber and the mechanism by which looping partners can stably find each other. Cohesin itself poses comparable mysteries (Fig. 3): how is this nonspecific DNA binder kept in place at both anchor sequences? If the complex indeed embraces and keeps together two distal cis-linked sequences, what, if any, is the trigger to open and close the ring? How dynamic is such a conformation? Tissue-invariant enhancer-promoter loops are an enigma particularly because enhancers otherwise show highly tissue-restricted activity. How can these regulatory DNA elements be involved in specific long-range DNA interactions when “inactive”? As we have discussed, CTCF or related factors might bind and topologically bookmark these sites. Besides CTCF, there are many transcription factors, often tissue-specific, that shape the genome and play roles not only in the formation of enhancer-promoter loops but also in the higher-order positioning of TADs. One would expect that they can change topology through self-associating domains, but, in many cases, this remains to be demonstrated. Alternatively or additionally, histone modifications could provide the “Velcro” that is necessary to keep distant sequences together. With CRISPR-Cas9 technology now at hand, and the ability therefore to manipulate any site in the genome and/or target any factor to a given genomic location, we expect that many of these questions will soon be addressed.

## Abbreviations

GR: glucocorticoid receptor
PcG: polycomb group
TAD: topologically associating domain
